# *FMR1* genetically interacts with *DISC1* to regulate glutamatergic synaptogenesis

**DOI:** 10.1038/s41537-024-00532-7

**Published:** 2024-11-27

**Authors:** Takato Honda, Kazuki Kurita, Yuko Arai, Himani Pandey, Akira Sawa, Katsuo Furukubo-Tokunaga

**Affiliations:** 1 The Picower Institute for Learning and Memory, Department of Brain and Cognitive Sciences, Massachusettes Institute of Technology (MIT), Cambridge, MA USA; 2 Stanley Center for Psychiatric Research, Broad Institute of MIT and Harvard, Cambridge, MA USA; 3 Department of Anesthesia, Critical Care, and Pain Medicine, Massachusettes General Hospital, Harvard Medical School, Boston, MA USA; 4 Life and Environmental Sciences, University of Tsukuba, Tsukuba, Japan; 5 Department of Biotechnology, Mahatma Gandhi Central University, Motihari, Bihar India; 6 Departments of Psychiatry, Neuroscience, Mental Health, Pharmacology, Biomedical Engineering and Genetic Medicine, Johns Hopkins University School of Medicine and Bloomberg School of Public Health, Johns Hopkins Medicine, Baltimore, MD USA

**Keywords:** Developmental biology, Genetics of the nervous system, Diseases of the nervous system

## Abstract

Synaptic development and functions have been hypothesized as crucial mechanisms of diverse neuropsychiatric disorders. Studies in past years suggest that mutations in the *fragile X mental retardation 1 (FMR1)* are associated with diverse mental disorders including intellectual disability, autistic spectrum disorder, and schizophrenia. In this study, we have examined genetical interactions between a select set of risk factor genes using fruit flies to find that *dfmr1*, the *Drosophila* homolog of the human *FMR1* gene, exhibits functional interactions with *DISC1* in synaptic development. We show that *DISC1* overexpression in the *dfmr1*^null^ heterozygous background causes synaptic alterations at the larval neuromuscular junctions that are distinct from those in the wild-type background. Loss of *dfmr1* modifies the *DISC1* overexpression phenotype in synaptic formation, suppressing the formation of synapse boutons. Interaction between the two genes was further supported molecularly by the results that *dfmr1* mutations suppress the *DISC1-*mediated upregulations of the postsynaptic expression of a glutamate receptor and the expression of ELKS/CAST protein, Bruchpilot, in presynaptic motoneurons. Moreover, *DISC1* overexpression in the *dfmr1*^null^ heterozygous background causes downregulation of a MAP1 family protein, Futsch. These results thus suggest an intriguing converging mechanism controlled by *FMR1* and *DISC1* in the developing glutamatergic synapses.

## Introduction

Schizophrenia is a severe mental disorder that impacts about 1% of the population^1–3^. Although the molecular and pathological mechanism of schizophrenia remains elusive, genome-wide and familial lineage studies of schizophrenia patients indicate multiple genetic risk factors contributing to the pathological condition. An increasing number of potential risk factor loci have been identified to date^4–15^. These studies also indicate that numerous genetic risk loci associated with schizophrenia are shared with other psychiatric disorders, including bipolar disorder, autism spectrum disorder, and intellectual disability^16–20^. Many of these shared risk loci encode genes for synaptic proteins, indicating a convergence in their biological functions toward pathways that regulate synaptic development and plasticity^9,10,14,21–23^. The molecular methods used to study schizophrenia have shown changes in a set of transcripts that control synaptic functions, which aligns well with the findings of genetic studies^24–26^. This further supports the synaptic hypothesis, suggesting that disrupted neuronal connectivity and signaling could be key causes of brain dysfunctions in patients^2,3,27^.

Fragile X syndrome is one of the most prevalent forms of intellectual disability and autistic abnormality^28–31^. Molecular studies have shown that fragile X syndrome (FXS) is caused by loss-of-functions of the *Fragile X Mental Retardation 1* (*FMR1)* gene, which encodes an RNA-binding protein (FMRP) that controls the translation of diverse synaptic proteins^32–36^. Loss of functions of *FMR1* results in defects in synaptic plasticity and cognition, with delayed dendritic spine maturation in both patients and knockout mice^28,29,31^. Researchers pre-synaptically induced an *FMR1* missense mutation, found in an FXS patient, to the *Drosophila* model and identified NMJ alterations in their branching and length^37^. In mice, an electrophysiological study revealed that the presynaptic *Fmr1* genotype influenced the degree of synaptic connectivity^38^. Moreover, genetical studies have shown that mutations of components of the FMRP complex are found among the de novo mutations enriched in schizophrenia patients^9,14,23^, suggesting an intriguing overlapping mechanism controlled by *FMR1* and schizophrenia risk genes.

The larval neuromuscular junction (NMJ) of the fruit fly (*Drosophila melanogaster*) shares several important characteristics with the excitatory synapses found in the vertebrate brain^39–43^. The NMJ fly uses glutamate as its primary transmitter and contains ionotropic glutamate receptors that are homologous to those found in humans^40,41,43^. Furthermore, similar to the vertebrate central synapse, the synapses at the fruit fly NMJ show dynamic plasticity with an organized series of boutons that are formed or eliminated during development and plasticity^39,40,42^. The fly NMJ’s stereotypic synaptic connections, with distinct and identifiable presynaptic motoneurons and postsynaptic muscles, make this system highly valuable for investigating the molecular genetic mechanisms of synaptogenesis and functions^40,42,44,45^.

As a way to analyze interactions between diverse psychiatric risk factor genes in synaptogenesis, we introduced the human *Disrupted-in-Schizophrenia-1 (DISC1)* gene in fruit flies. A balanced chromosomal translocation affecting *DISC1* locus was initially identified in a large Scottish family with a variety of mental disorders, including schizophrenia, major depression, bipolar disorder, and autism spectrum disorder^46–51^. We previously showed that overexpression of *DISC1* (*DISC1*^*OE*^) causes anatomical alteration of synaptic structures in the larval NMJ, resulting in suppression of the total bouton area^52,53^. Based on this finding, we have genetically screened other schizophrenia risk factor genes for functional interactions with *DISC1* in the developing glutamatergic synapses and identified several interacting genes, including *dfmr1*, the fruit fly *FMR1* homolog^30,54–59^. It has been reported that the ortholog of the vertebrate FMRP (dFMRP) in fruit fly NMJ is expressed in both presynaptic motor neurons and in postsynaptic muscles in larvae^59^. In the present study, we show that loss of *dfmr1* modifies the *DISC1*^*OE*^ phenotype in synaptogenesis, suppressing the formation of synapse boutons. We also demonstrate that *dfmr1* mutations suppress the *DISC1-*mediated upregulations of the expressions of a glutamate receptor (DGluRIIA)^60–62^ in postsynaptic cells and a ELKS/CAST protein, Bruchpilot (Brp)^63–66^ in presynaptic cells. Moreover, *DISC1* overexpression in the *dfmr1*^null^*/+* background causes downregulation of a MAP1 family protein, Futsch^56,59,67–70^. These results suggest an intriguing converging mechanism controlled by *FMR1* and *DISC1* in the developing glutamatergic synapses.

## Results

### Genetic screening of *DISC1* interactors in synaptogenesis

**Fig. 1:**
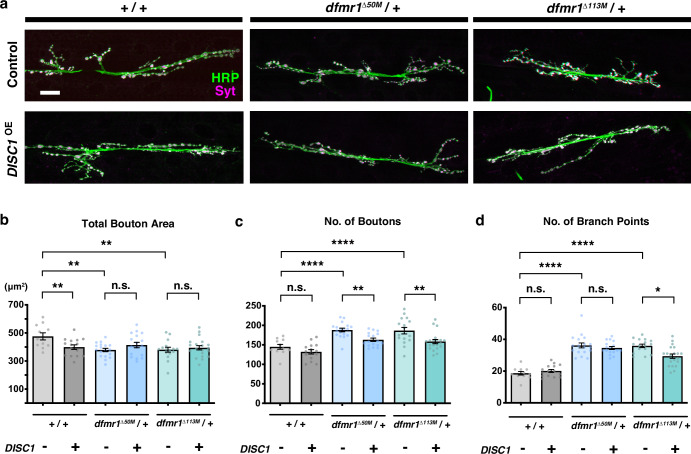
*dfmr1* interacts with *DISC1* in synaptogenesis. **a** Larval NMJs with DISC1 expression in *dfmr1*^null^*/+* backgrounds. NMJs of muscles 6-7 in the second abdominal segment were stained with anti-HRP (green) and anti-synaptotagmin (magenta). Bar, 20 μm. HRP: horseradish peroxidase protein. Syt: synaptotagmin. **b** Quantification of the NMJ morphology of the total bouton area (μm^2^) in heterozygous *dfmr1*^null^*/+* (*dfmr1*^Δ50M^ or *dfmr1*^Δ113M^) larvae with (+) or without (−) DISC1 overexpression (*DISC1*^OE^). One-way ANOVA (*F* (5, 91) = 3.417, *p* = 0.0071) followed by Holm–Sidak’s multiple comparisons (*w (CS10)*; *DISC1*^OE^ (−) vs. *w (CS10)*; *DISC1*^OE^ (+), *p* = 0.0185, *t* = 2.802, *df* = 91. *w (CS10)*; *DISC1*^OE^ (−) vs. *dfmr1*^Δ50M^; *DISC1*^OE^ (−), *p* = 0.0019, *t* = 3.693, *df* = 91. *w (CS10)*; *DISC1*^OE^ (−) vs. *dfmr1*^Δ113M^; *DISC1*^OE^ (−), *p* = 0.0026, *t* = 3.534, *df* = 91. *dfmr1*^Δ50M^; *DISC1*^OE^ (−) vs. *dfmr1*^Δ50M^; *DISC1*^OE^ (+), *p* = 0.2434, *t* = 1.527, *df* = 91. *dfmr1*^Δ113M^; *DISC1*^OE^ (−) vs. *dfmr1*^Δ113M^; *DISC1*^OE^ (+), *p* = 0.5506, *t* = 0.5991, *df* = 91). **c** Quantification of the NMJ morphology of the number of boutons in heterozygous *dfmr1*^null^*/+* (*dfmr1*^Δ50M^ or *dfmr1*^Δ113M^) larvae with (+) or without (−) DISC1 overexpression (*DISC1*^OE^). One-way ANOVA (*F* (5, 91) = 14.17, *p* < 0.0001) followed by Holm–Sidak’s multiple comparisons (*w (CS10)*; *DISC1*^OE^ (−) vs. *w (CS10)*; *DISC1*^OE^ (+), *p* = 0.1730, *t* = 1.374, *df* = 91. *w (CS10)*; *DISC1*^OE^ (−) vs. *dfmr1*^Δ50M^; *DISC1*^OE^ (−), *p* < 0.0001, *t* = 4.856, *df* = 91. *w (CS10)*; *DISC1*^OE^ (−) vs. *dfmr1*^Δ113M^; *DISC1*^OE^ (−), *p* < 0.0001, *t* = 4.561, *df* = 91. *dfmr1*^Δ50M^; *DISC1*^OE^ (−) vs. *dfmr1*^Δ50M^; *DISC1*^OE^ (+), *p* = 0.0040, *t* = 3.184, *df* = 91. *dfmr1*^Δ113M^; *DISC1*^OE^ (−) vs. *dfmr1*^Δ113M^; *DISC1*^OE^ (+), *p* = 0.0016, *t* = 3.591, *df* = 91). **d** Quantification of the NMJ morphology of the number of branch points in heterozygous *dfmr1*^null^*/+* (*dfmr1*^Δ50M^ or *dfmr1*^Δ113M^) larvae with (+) or without (−) DISC1 overexpression (*DISC1*^OE^). Kruskal–Wallis test (*p* < 0.0001) followed by Dunn’s multiple comparisons (*w (CS10)*; *DISC1*^OE^ (−) vs. *w (CS10)*; *DISC1*^OE^ (+), *p* > 0.9999, *z* = 0.2538. *w (CS10)*; *DISC1*^OE^ (−) vs. *dfmr1*^Δ50M^; *DISC1*^OE^ (−), *p* < 0.0001, *z* = 5.101. *w (CS10)*; *DISC1*^OE^ (−) vs. *dfmr1*^Δ113M^; *DISC1*^OE^ (−), *p* < 0.0001, *z* = 5.141. *dfmr1*^Δ50M^; *DISC1*^OE^ (−) vs. *dfmr1*^Δ50M^; *DISC1*^OE^ (+), *p* > 0.9999, *z* = 0.4182. *dfmr1*^Δ113M^; *DISC1*^OE^ (−) vs. *dfmr1*^Δ113M^; *DISC1*^OE^ (+), *p* = 0.0462, *z* = 2.603). **b**–**d** Significance levels in the figures are represented as *p* < 0.05 (*), *p* < 0.01 (**), *p* < 0.001 (***), *p* < 0.0001 (****), and n.s. not significant. *n* = 11–20. Individual values are plotted in the graphs. Data are presented as the mean ± SEM.

To investigate interactions of *DISC1* and other risk factor genes in a genetically tractable model, we overexpressed *DISC1* in the fruit fly NMJ using the *GAL4*-*UAS* system. The overexpression of *DISC1* (*DISC1*^OE^) with a ubiquitous *tubP-GAL4* driver^71^ caused a significant reduction in the total synaptic bouton area, although the numbers of synaptic boutons and axonal branch points were not altered (Fig. 1a–c, comparisons of *w +/+* data between *DISC1*^OE^ − and *+*). To examine spatial requirement, we also overexpressed *DISC1* specifically in the pre- and postsynaptic cells using a motoneuron-specific *elav-GAL4* driver^72^, or a muscle-specific *C57*-*GAL4* driver^73^, respectively. Neither pre- nor postsynaptic overexpression altered synaptic formation (Supplementary Fig. S1a–c, comparisons of *w +/+* data between *DISC1* with *+* or without − overexpression), indicating that simultaneous overexpression in both pre- and postsynaptic cells was necessary for the anatomical alteration. To identify risk factor genes that interact with *DISC1* in synaptogenesis, we set up a genetic crossing (Supplementary Fig. S2) between the *DISC1*^OE^ flies (hereafter *DISC1*^OE^ represents the overexpression of *DISC1* under *tubP-GAL4* driver) and the fly mutants of diverse schizophrenia risk genes (Supplementary Table S1). In this study, we used the mutations that varied from null to hypomorphic mutants, resulting in a maximum reduction of ~50% in the gene dosage when present in the heterozygous condition used in this screening. They only have a partial impact on synaptic formations when acting independently. To evaluate genetic modifications, we examined the synaptic anatomy of NMJ, focusing on the three morphological parameters (bouton area, number of boutons, and number of axonal branch points), consistent with the previously established methods^52,53^. We analyzed mutations found on the fly autosomes (second and third chromosomes). Despite analyzing a small number of mutations, we identified several genes, such as *dfmr1*, which is the fruit fly homolog of the human *FMR1* gene^30,54–59^.

### *dfmr1* genetically interacts with *DISC1* in synaptogenesis

We analyzed two *dfmr1* null mutations (*dfmr1*^Δ50M^ and *dfmr1*^Δ113M^)^59^ and found that both mutations on their own caused suppression of the total bouton area, an increase in the number of synaptic boutons, and an increase in the number of the axonal branch points in the *dfmr1*^null^*/+* heterozygous animals (Fig. 1a–d) (comparisons of *DISC1*^OE^ − data between *w +/+* and *dfmr1*^null^*/+*). Whereas *DISC1*^OE^ suppressed the total bouton area in the *w (CS10)* control background (*+/+*) (Fig. 1b) (comparison of *w +/+* data between *DISC1*^OE^ − and *+*), no difference was caused by *DISC1*^OE^ in the *dfmr1*^null^*/+* heterozygous backgrounds (Fig. 1b) (comparisons of *dfmr1*^null^*/+* data between *DISC1*^OE^ − and *+*). On the other hand, *DISC1*^OE^ caused reductions in the number of synaptic boutons in the *dfmr1*^null^*/+* heterozygous backgrounds (Fig. 1c) (comparisons of *dfmr1*^null^*/+* data between *DISC1*^OE^ − and *+*), while it resulted in no difference in the wild-type background (Fig. 1c) (comparison of *w +/+* data between *DISC1*^OE^ − and *+*). Although *DISC1*^OE^ displayed no alteration in the number of axonal branch points in the wild type (Fig. 1d) (comparison of *w +/+* data between *DISC1*^OE^ − and *+*), there was a moderate but significant reduction (*p* = 0.046) in the number of branch points in the *dfmr1*^Δ113M^ heterozygous background, but not in the *dfmr1*^Δ50M^ (Fig. 1d) (comparisons of *dfmr1*^null^*/+* data between *DISC1*^OE^ − and *+*).

To confirm the modifications of the *DISC1*^OE^ phenotypes by *dfmr1*, we knocked down the expression of *dfmr1* using RNA interference (RNAi). Intriguingly, overexpression of *DISC1* in *dfmr1* RNAi caused pupal mortality. All the *dfmr1* RNAi flies with *DISC1*^OE^ died during the pupal stage (*n* = 53), while none of the *dfmr1* RNAi animals without *DISC1*^OE^ exhibited such mortality, eventually developing into adult flies (*n* = 47).

**Fig. 2:**
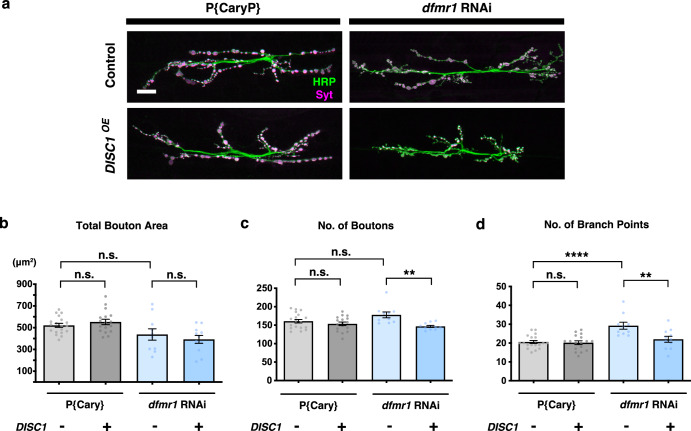
Genetic modification of *DISC1* phenotype by *dfmr1* RNAi. **a** *P{Cary P}*: a fly line with an empty vector inserted at the same genetic locus as the RNAi line. *dfmr1* RNAi: driven by *tubP-GAL4*. NMJs of muscles 6-7 in the second abdominal segment were stained with anti-HRP (green) and anti-synaptotagmin (magenta). Bar, 20 μm. **b** Quantification of the NMJ morphology of the total bouton area (μm^2^) in *dfmr1* RNAi larvae with (+) or without (−) DISC1 overexpression (*DISC1*^OE^). One-way ANOVA (*F* (3, 53) = 5.671, *p* = 0.0019) followed by Holm–Sidak’s multiple comparisons (*P{Cary P}*; *DISC1*^OE^ (−) vs. *P{Cary P}*; *DISC1*^OE^ (+), *p* = 0.6035, *t* = 0.8296, *df* = 53. *P{Cary P}*; *DISC1*^OE^ (−) vs. *dfmr1*^RNAi^; *DISC1*^OE^ (−), *p* = 0.1757, *t* = 1.904, *df* = 53. *dfmr1*^RNAi^; *DISC1*^OE^ (−) vs. *dfmr1*^RNAi^; *DISC1*^OE^ (+), *p* = 0.6035, *t* = 0.9036, *df* = 53). **c** Quantification of the NMJ morphology of the number of boutons in *dfmr1* RNAi larvae with (+) or without (−) DISC1 overexpression (*DISC1*^OE^). Kruskal–Wallis test (*p* < 0.0119) followed by Dunn’s multiple comparisons (*P{Cary P}*; *DISC1*^OE^ (−) vs. *P{Cary P}*; *DISC1*^OE^ (+), *p* > 0.9999, *z* = 0.8461. *P{Cary P}*; *DISC1*^OE^ (−) vs. *dfmr1*^RNAi^; *DISC1*^OE^ (−), *p* = 0.2366, *z* = 1.757. *dfmr1*^RNAi^; *DISC1*^OE^ (−) vs. *dfmr1*^RNAi^; *DISC1*^OE^ (+), *p* = 0.0043, *z* = 3.186). **d** Quantification of the NMJ morphology of the number of branch points in *dfmr1* RNAi larvae with (+) or without (−) DISC1 overexpression (*DISC1*^OE^). One-way ANOVA (*F* (3, 53) = 10.10, *p* < 0.0001) followed by Holm–Sidak’s multiple comparisons (*P{Cary P}*; *DISC1*^OE^ (−) vs. *P{Cary P}*; *DISC1*^OE^ (+), *p* = 0.7892, *t* = 0.2687, *df* = 53. *P{Cary P}*; *DISC1*^OE^ (−) vs. *dfmr1*^RNAi^; *DISC1*^OE^ (−), *p* < 0.0001, *t* = 4.919, *df* = 53. *dfmr1*^RNAi^; *DISC1*^OE^ (−) vs. *dfmr1*^RNAi^; *DISC1*^OE^ (+), *p* = 0.0011, *t* = 3.673, *df* = 53). **b**–**d** Significance levels in the figures are represented as *p* < 0.01 (**), *p* < 0.0001 (****), and n.s. not significant. *n* = 10–19. Individual values are plotted in the graphs. Data are presented as the mean ± SEM.

Because of the difference in the genetic background, the *DISC1*^OE^ phenotype with the control *P{CaryP}* larvae was different from that in the *w (CS10)* control larvae. Synaptic development was affected by *dfmr1* RNAi on its own, exhibiting an increase only in the number of the branch points (Fig. 2a–d) (comparisons of *DISC1*^OE^ − data between *P{CaryP}* and *dfmr1 RNAi*). *DISC1*^OE^ failed to cause anatomical alteration of the bouton areas in the *P{CaryP}* control background. Since it has been reported that genetic background can be a variable factor affecting NMJ structures^74^, this difference is potentially because of the genetic background between *P{CaryP}* and *w (CS10)*. On the other hand, *DISC1*^OE^ caused a significant decrease in the numbers of the synaptic boutons and the axonal branch points with *dfmr1* RNAi (Fig. 2c, d) (comparisons of *dfmr1 RNAi* data between *DISC1*^OE^ − and *+*), consistent with the results in *dfmr1*^null^*/+* mutants.

Overall, the NMJ anatomical results show that DISC1 overexpression in the *dfmr1*^null^*/+* heterozygous background causes synaptic alterations at the larval NMJs, potentially by convergent regulatory networks. To further understand the morphological phenotypes at the molecular level, we next investigated the protein expression levels of potential interactors of *DISC1* and *FMR1* in synaptogenesis.

### *dfmr1* suppresses *DISC1*-mediated DGluRIIA and Brp stimulations

FMRP is essential in the translational regulation of various synaptic proteins^32–36^. To further investigate the genetic interaction between *dfmr1* and *DISC1* at the molecular level, we analyzed the expressions of DGluRIIA, reported as one of the key targets of FMRP-mediated translational regulation in the synapses^59,75^. DGluRIIA is one of the five AMPA-like receptor subunits (DGluRIIA–DGluRIIE), which are expressed post-synaptically in the fly muscle^60–62^.

**Fig. 3:**
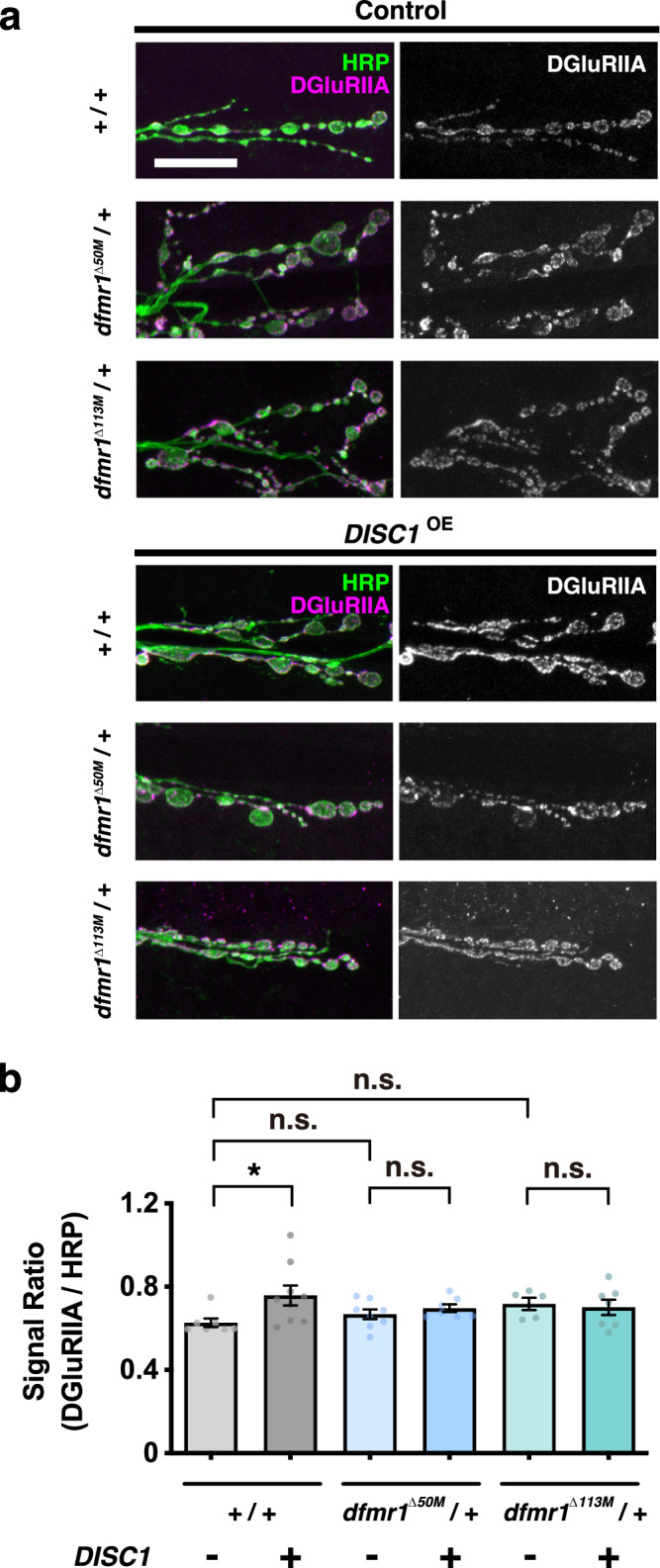
DGluRIIA expression in the control and *DISC1*^OE^ NMJs. **a** NMJs of muscles 6-7 in the second abdominal segment were stained with anti-HRP (green) and anti-DGluRIIA (magenta). Bar, 20 μm. **b** Quantification of DGluRIIA protein expression level normalized to HRP immunoreactivity in *dfmr1*^null^*/+* (*dfmr1*^Δ50M^ or *dfmr1*^Δ113M^) NMJ boutons with (+) or without (−) DISC1 overexpression (*DISC1*^OE^). Kruskal–Wallis test (*p* = 0.0832) followed by Dunn’s multiple comparisons (*w (CS10)*; *DISC1*^OE^ (−) vs. *w (CS10)*; *DISC1*^OE^ (+), *p* = 0.0372, *z* = 2.677. *w (CS10)*; *DISC1*^OE^ (−) vs. *dfmr1*^Δ50M^; *DISC1*^OE^ (−), *p* = 0.8535, *z* = 1.370. *w (CS10)*; *DISC1*^OE^ (−) vs. *dfmr1*^Δ113M^; *DISC1*^OE^ (−), *p* = 0.0661, *z* = 2.478. *dfmr1*^Δ50M^; *DISC1*^OE^ (−) vs. *dfmr1*^Δ50M^; *DISC1*^OE^ (+), *p* > 0.9999, *z* = 0.8286. *dfmr1*^Δ113M^; *DISC1*^OE^ (−) vs. *dfmr1*^Δ113M^; *DISC1*^OE^ (+), *p* > 0.9999, *z* = 0.7481). Significance levels in the figures are represented as *p* < 0.05 (*) and n.s. not significant. *n* = 5–9. Individual values are plotted in the graphs. Data are presented as the mean ± SEM.

In this study, we found that *DISC1*^OE^ stimulated the DGluRIIA level in the *+/+* control background (Fig. 3a, b) (comparison of *w +/+* data between *DISC1*^OE^ − and *+*). On the other hand, there was no alteration detected in DGluRIIA level in the heterozygous *dfmr1*^null^/+ backgrounds on its own (Fig. 3b) (comparisons of *DISC1*^OE^ − data between *w* +/+ and *dfmr1*^null^/+). In addition, *DISC1*^OE^ did not change DGluRIIA level in *dfmr1*^null^ mutants (Fig. 3b) (comparisons of *dfmr1*^null^/+ between *DISC1*^OE^ − and +). These results indicated that both *dfmr1* mutations suppressed DISC1-mediated stimulation of DGluRIIA expression, suggesting the dfmr1 activity mediates DGluRIIA stimulation by *DISC1*^OE^.

**Fig. 4:**
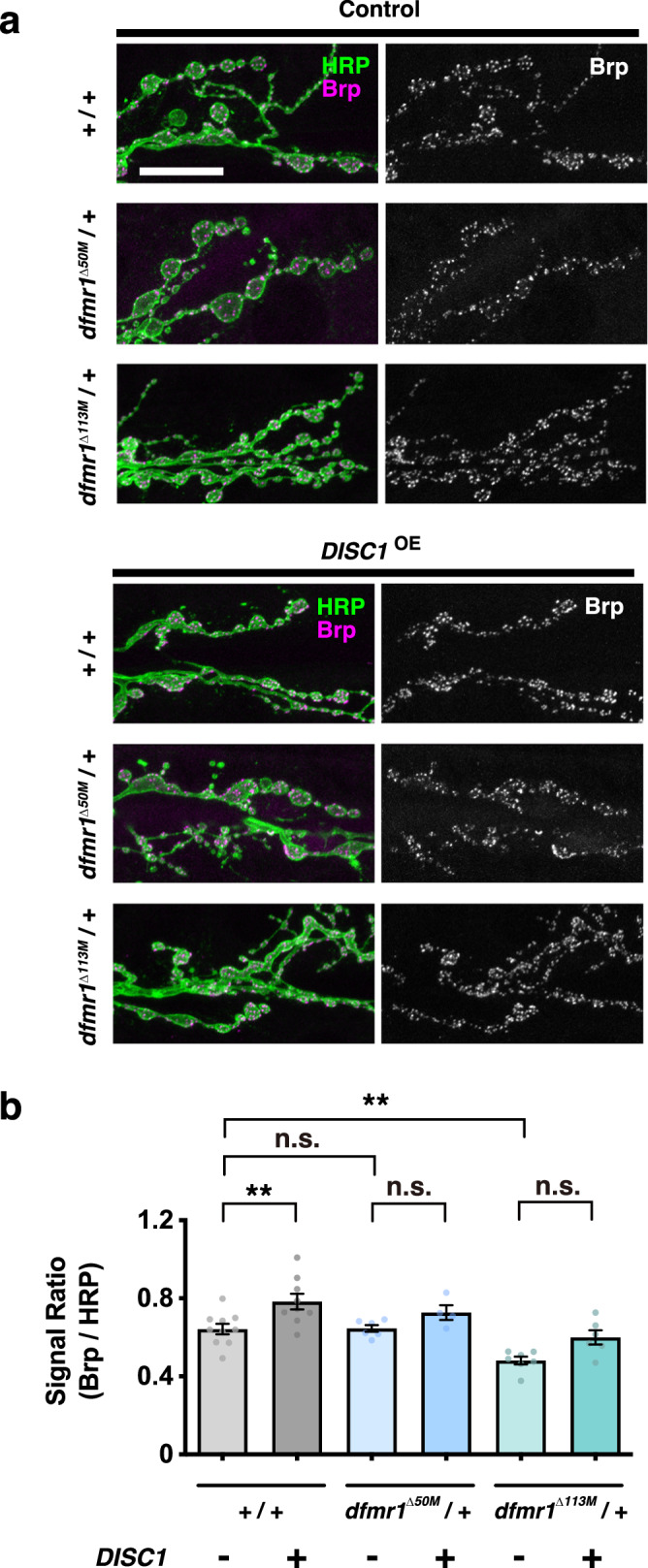
Brp expression in the control and *DISC1*^OE^ NMJs. **a** NMJs of muscles 6-7 in the second abdominal segment were stained with anti-HRP (green) and anti-Brp (magenta). Bar, 20 μm. **b** Quantification of Brp protein expression level normalized to HRP immunoreactivity in *dfmr1*^null^*/+* (*dfmr1*^Δ50M^ or *dfmr1*^Δ113M^) NMJ boutons with (+) or without (−) DISC1 overexpression (*DISC1*^OE^). One-way ANOVA (*F* (5, 36) = 10.94, *p* < 0.0001) followed by Holm–Sidak’s multiple comparisons (*w (CS10)*; *DISC1*^OE^ (−) vs. *w (CS10)*; *DISC1*^OE^ (+), *p* = 0.0039, *t* = 3.591, *df* = 36. *w (CS10)*; *DISC1*^OE^ (−) vs. *dfmr1*^Δ50M^; *DISC1*^OE^ (−), *p* = 0.9395, *t* = 0.0764, *df* = 36. *w (CS10)*; *DISC1*^OE^ (−) vs. *dfmr1*^Δ113M^; *DISC1*^OE^ (−), *p* = 0.0025, *t* = 3.828, *df* = 36. *dfmr1*^Δ50M^; *DISC1*^OE^ (−) vs. *dfmr1*^Δ50M^; *DISC1*^OE^ (+), *p* = 0.2730, *t* = 1.481, *df* = 36. *dfmr1*^Δ113M^; *DISC1*^OE^ (−) vs. *dfmr1*^Δ113M^; *DISC1*^OE^ (+), *p* = 0.0509, *t* = 2.497, *df* = 36). Significance levels in the figures are represented as *p* < 0.01 (**) and n.s. not significant. *n* = 4–10. Individual values are plotted in the graphs. Data are presented as the mean ± SEM.

To further investigate the functional interaction between *dfmr1* and *DISC1* in synaptogenesis at high resolution, we used the presynaptic marker Bruchpilot (Brp), which is the fly homolog of the vertebrate ELKS/CAST active zone proteins and is crucial for rapid synaptic vesicle release^63–66^. *DISC1*^*OE*^ caused significant increases in the Brp level in the wild-type background (Fig. 4a, b) (comparisons of *w +/+* data between *DISC1*^OE^ − and *+*). On the other hand, *DISC1*^OE^ did not change the Brp level in *dfmr1*^null^/+ mutants (Fig. 4b) (comparisons of *dfmr1*^null^/+ between *DISC1*^OE^ − and +), suggesting *dfmr1* mutations suppressed DISC1-mediated stimulation of Brp expression. These results suggest that *dfmr1* suppresses DISC1-mediated DGluRIIA and Brp stimulations.

### DISC1 suppresses the MAP1 homolog in *dfmr1* background

**Fig. 5:**
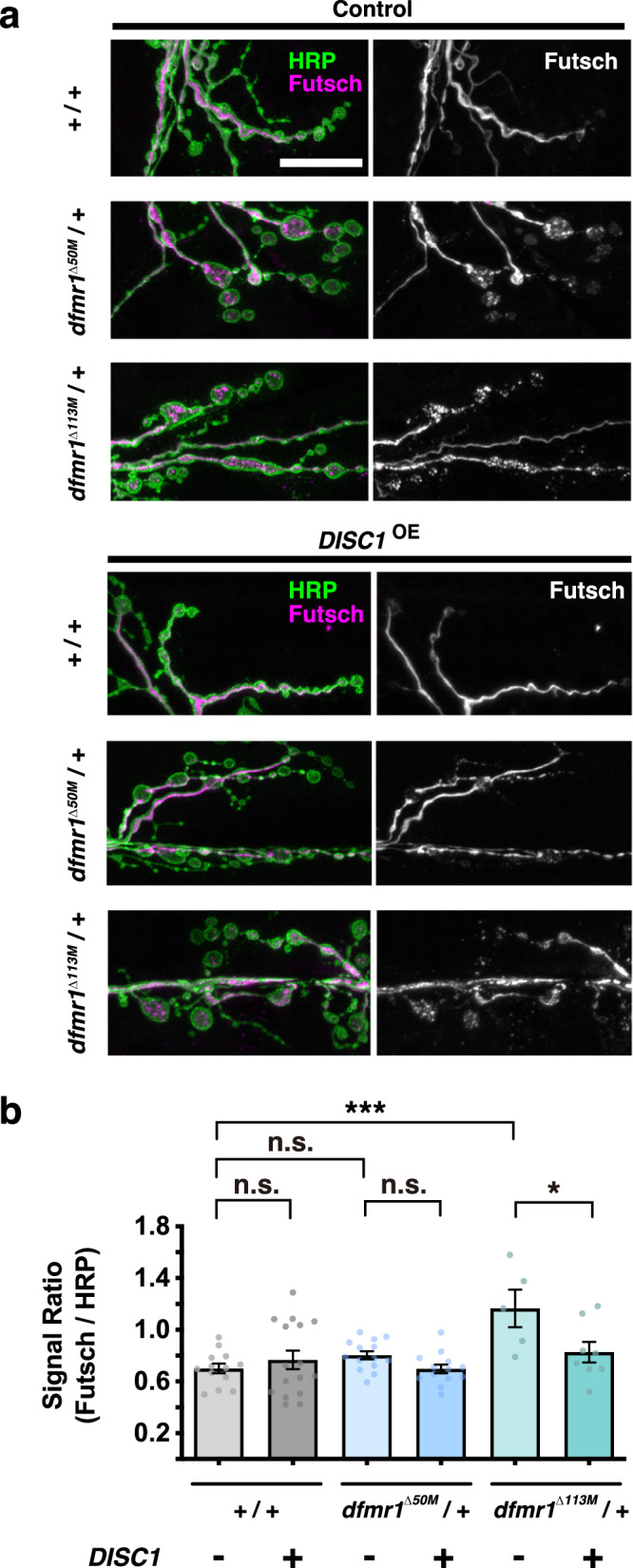
Futsch expression in the control and *DISC1*^OE^ NMJs. **a** NMJs of muscles 6-7 in the second abdominal segment were stained with anti-HRP (green) and anti-Futsch (magenta). Bar, 20 μm. **b** Quantification of Futsch protein expression level normalized to HRP immunoreactivity in *dfmr1*^null^*/+* (*dfmr1*^Δ50M^ or *dfmr1*^Δ113M^) NMJ boutons with (+) or without (−) DISC1 overexpression (*DISC1*^OE^). One-way ANOVA (*F* (5, 63) = 4.620, *p* = 0.0012) followed by Holm–Sidak’s multiple comparisons (*w (CS10)*; *DISC1*^OE^ (−) vs. *w (CS10)*; *DISC1*^OE^ (+), *p* = 0.4444, *t* = 0.8837, *df* = 63. *w (CS10)*; *DISC1*^OE^ (−) vs. *dfmr1*^Δ50M^; *DISC1*^OE^ (−), *p* = 0.4444, *t* = 1.324, *df* = 63. *w (CS10)*; *DISC1*^OE^ (−) vs. *dfmr1*^Δ113M^; *DISC1*^OE^ (−), *p* = 0.0002, *t* = 4.380, *df* = 63. *dfmr1*^Δ50M^; *DISC1*^OE^ (−) vs. *dfmr1*^Δ50M^; *DISC1*^OE^ (+), *p* = 0.4444, *t* = 1.362, *df* = 63. *dfmr1*^Δ113M^; *DISC1*^OE^ (−) vs. *dfmr1*^Δ113M^; *DISC1*^OE^ (+), *p* = 0.0182, *t* = 2.940, *df* = 63). Significance levels in the figures are represented as *p* < 0.05 (*), *p* < 0.001 (***), and n.s. not significant. *n* = 5–16. Individual values are plotted in the graphs. Data are presented as the mean ± SEM.

We then analyzed expression levels of another synaptic protein, the *Drosophila* homolog of the MAP1 family proteins, Futsch^56,59,69,70^. Futsch is a key presynaptic protein essential for the development of synaptic microtubule cytoskeletons^70,76,77^. Consistent with the fact that Futsch is negatively regulated by dFMRP^56,59^, *dfmr1*^Δ113M^/+ caused upregulation of Futsch on their own (Fig. 5a, b) (comparisons of *DISC1*^OE^ − data between *w* +/+ and *dfmr1*^null^/+). *DISC1*^OE^ caused no alteration in the Futsch level in the *+/+* control background (Fig. 5b) (comparison of *w +/+* data between *DISC1*^OE^ − and *+*). On the other hand, *DISC1*^OE^ downregulated the Futsch level in the *dfmr1*^Δ113M^/+ heterozygous background (Fig. 5b) (comparisons of *dfmr1*^Δ113M^/+ between *DISC1*^OE^ − and +). This result supports functional interaction between *DISC1* and *dfmr1* in synaptic development.

## Discussion

The development and plasticity of synapses have been hypothesized as one of the critical mechanisms of diverse psychiatric disorders^9,10,14,21,22^. In this study, we have found that *dfmr1*, the *Drosophila* homolog of *FMR1*, exhibits functional interactions with *DISC1* in synaptic development. We have shown that mutations of *dfmr1* modify the *DISC1*^OE^ synaptic phenotypes at the molecular and morphological levels. In summary, the various synaptic phenotypes were uniquely detected in the presence of both *DISC1*^OE^ and *dfmr1*^null^ heterozygous mutations (*DISC1*^OE^; *dfmr1*^null^*/+*), which includes, for the NMJ morphology, (1) neutralization of decreased bouton area induced individually by *DISC1*^OE^ and *dfmr1*^null^*/+*, (2) decreased numbers of boutons and branches (no change in *DISC1*^OE^ only). For the protein expression level, the *DISC1*^OE^; *dfmr1*^null^*/+* mutants displayed (3) neutralization of increased DGluRIIA and Brp levels induced by *DISC1*^OE^, and (4) decreased Brp expression level (no change in *DISC1*^OE^ only). As a limitation of the study, since we have not been able to show a direct interaction between the FMRP and DISC1 proteins in our model, it is still difficult to provide a definitive conclusion for the questions such as (a) whether the above phenotypes are the outcomes of their direct or indirect interactions, (b) whether *DISC1* and *dfmr1* function in the same pathway or multiple pathways are involved in this process. It is noteworthy that the molecular studies on the DISC1 interacting proteins so far have failed to identify FMRP among the members of the direct interactome^47,50,78–80^. These results as a whole suggest that the genetical interaction of *DISC1* and *dfmr1* may reflect complex convergence of regulatory networks in the developing glutamatergic synapses rather than direct regulatory cascades. As the next research directions, we discuss the potential overlapping regulatory networks of DISC1 and FMRP in synaptic development and plasticity in the following sections.

As a potential molecular pathway that links DISC1 and FMRP, it has been known that DISC1 inhibits glycogen synthase kinase-3 (GSK3β) activity through direct physical interaction^81^. In *Fmr1*^KO^ mice, the GSK3β activity level was elevated^82^, and the GSK3β inhibition improved hippocampus-dependent learning and rescued neurogenesis^83^. GSK3β is also known to phosphorylate FXS-related protein 1 (FXR1), an FMRP-interacting protein. GSK3β-FXR1 pathway is known to play a role in glutamatergic neurotransmission^84^. Future studies focusing on DISC1/GSK3β/FMRP in the context of glutamatergic synaptogenesis will have the potential to be further investigated.

Defects in synaptic plasticity and cognition occur due to the loss of functions of *FMR1*, leading to delayed dendritic spine maturation in both patients and knockout mice^28,29,31^. dFMRP in fruit fly NMJ is expressed pre-synaptically in the motor neurons and post-synaptically in the muscle^59^. We have shown that overexpression of *DISC1* in pre- and postsynaptic cells is required for anatomical alterations of the NMJ synapses, suggesting that *dfmr1* interacts with *DISC1*^OE^ to regulate both pre- and postsynaptic regulatory processes.

FMRP has been known to control glutamate release and other anterograde signaling, such as Wnt and Jeb^45^, as well as in the presynaptic termini^55^. In the postsynaptic cells, it has been suggested that FMRP regulates synaptic development and plasticity by repressing the translation of specific mRNAs, which encode about 30% of the postsynaptic density (PSD) proteins such as glutamate-receptor interacting proteins^68^. Furthermore, FMRP downregulates the synthesis of cytoskeletal regulatory proteins, which includes MAP1B, P21-activated kinase 1 (PAK), and Ras-related C3 botulinum toxin substrate 1 (RAC1)^59,85–87^, as well as regulators of α-amino-3-hydroxyl-5-methyl-4-isoxazole-propionate (AMPA) receptors such as Activity-regulated Cytoskeleton-associated protein (ARC)^88,89^.

As with FMRP, various molecular studies have demonstrated the importance of DISC1 for synaptic development and plasticity^46–51,78–80^. *DISC1* encodes a protein with functional domains that interact with several proteins and play multiple roles in various biological processes^46–51,78–80^. In particular, it has been demonstrated that DISC1 localization to PSD is important for the regulation of spine morphology and AMPA-type glutamate receptor expression^79,80^. The series of studies of DISC1 interactome^46,47,50,51,78–80,90^ have identified the various synaptic proteins, including the PSD interacting proteins such as Kalirin7 (KAL7). In particular, DISC1 stimulates KAL7-PSD95 interaction to negatively regulate KAL7 association with RAC1. Additionally, DISC1 has been known to interact with Traf2 and Nck-interacting kinase (TNIK), which plays roles in the turnover of some PSD proteins, including the AMPA receptor subunits, GLUR1 and PSD95^80^. It has been shown that PAK inhibitors such as FARX486 ameliorate dendritic spine deterioration and behavioral deficits induced by the knockdown of DISC1^91^. Importantly, in *Fmr1* knockout mice, FRAX486 rescues dendritic spine defects, seizures, and behavioral abnormalities such as hyperactivity and repetitive movements^87^.

Therefore, these studies indicate a shared mechanism between FMRP and DISC1 in regulating the cytoskeleton and expressing glutamate receptor components in developing synapses. We have found that as many as 28% (29/103) of the proteins that exhibit direct association with DISC1 are found among the regulatory targets of FMRP (Supplementary Table S2). Notably, the majority of the genes (27/29) encoding the DISC1-interacting proteins targeted by FMRP are conserved in fruit flies in the context of the regulation of synaptic growth and plasticity (Supplementary Table S2).

In the present study, we showed the alternations of NMJ anatomical structures based on the total bouton areas, the number of boutons, and the number of axonal branching points. A comprehensive review^42^ suggested that multiple genes are differentially involved in each NMJ morphological phenotype, including the above three parameters. For example, *Ankyrin2* mutant flies displayed a decreased number of boutons but increased bouton size in NMJ^42^. In contrast, the NMJs of *Highwire* mutants showed an increased number of boutons but decreased bouton size^42^. *Highwire* knockdown increased the number of branch points^74^. Based on the accumulating knowledge about NMJ growth, each of the three phenotypes in our results (Figs. 1 and 2) potentially has independent molecular mechanisms for each component of NMJ morphology regulated by *DISC1* and *dfmr1*.

Although *dfmr1*^Δ113M^*/+* and *dfmr1*^Δ50M^*/+* displayed equivalent phenotypes in most cases in the present results, the difference in *dfmr1*^null^*/+* mutations, such as in the NMJ branch points (Fig. 1d) and Futsch expression level (Fig. 5b) are potentially related to the specific location of their deletion sites. Particularly, while both mutations are reported as functionally protein nulls^59^, the deletion site of *dfmr1*^Δ113M^ contains an additional coding region^59^, which may involve a difference in molecular interactions between *DISC1* and *dfmr1*. Considering the nature of FMRP as mRNA binding protein^92^, the missing coding region may affect the FMRP binding profile with its target mRNAs. Although we characterized the changes in protein expression levels upon *DISC1*^OE^; *dfmr1*^null^*/+* mutations, as a limitation of this study, it remains elusive how *DISC1*^OE^ is involved in FMRP RNA-binding activity and RNA stability. Beyond direct protein-protein interactions and regulations, FMRP has multiple functions in RNA metabolism, including translation, stability, editing, and intracellular transport^92^. Further research on profiling the mRNA binding patterns focusing on FMRP and DISC1 networks, such as comprehensive RNA-sequencing analysis with *DISC1* and *dfmr1* modifications, will provide further insight into the molecular basis of schizophrenia and the identification of future therapeutic targets. In addition to the studies at the RNA level, further investigations on the behavioral phenotypes in *DISC1*^OE^; *dfmr1* ^null^*/+* animals (e.g., locomotion^93^, associative learning^94,95^, and sleep^96,97^) will provide the multilayered understanding of the DISC1-FMR1 network at the systems level.

Previous studies^9,14,23^ have shown that schizophrenia patients have enriched de novo mutations in genes belonging to the postsynaptic density at glutamatergic synapses, such as components of the PSD complex, *N*-methyl-D-aspartic acid (NMDA) receptor signaling complex, ARC interactors, and the FMRP complex. It has been suggested that mutations in FMR1 are associated with diverse mental disorders, including intellectual disability, autistic abnormality, and schizophrenia^29–36^. Focusing on genetical interactions in synaptic development, our study highlights a common molecular underpinning regulated by *FMR1* and *DISC1* that contribute to the pathological process of neuropsychiatric disorders.

## Materials and methods

### Fly stocks

As the standard stock, a *white* (*w*) stock outcrossed with *Canton S* 10 times (*w (CS10)*) was used in the present study. The details of the construction of transgenic flies carrying *UAS-DISC1* transgene were described previously^52,53^. The fly stocks were outcrossed to *w (CS10)* at least 5 times to ensure a homogeneous genetic background. The following fly stocks were obtained from the Bloomington Stock Center (Bloomington, IN, USA): *dfmr1* null mutants (*dfmr1*^Δ50M^ and *dfmr1*^Δ113M^)^57,59^, *dfmr1* RNAi (*dfmr1*^HMS00248^) and the control *P{CaryP}* stocks, and *GAL4* drivers (*tubP-GAL4*^71^, *elav-GAL4*^72^, and *C57*-*GAL4*^73^). All stocks were raised at 25 °C on a standard fly food.

### Genetic screening

The details of the screening scheme were described in Supplementary Fig. S2 and previously^52,53^. The mutant lines were balanced with a double balancer stock (*w/w; Sp / CyO Act-GFP; Pr Dr/ TM6B ubi-GFP*) for this genetic screening. Then, the resulting progeny carrying a mutation were crossed either with control (*w; +; tubP-GAL4/TM6B ubi-GFP*) or with *DISC1*^*OE*^ (*w; UAS-DISC1(CS10)6-6(II); tubP-GAL4/ TM6B ubi-GFP*) flies. Larvae were raised at 25 °C, and non-GFP expressing flies were selected for dissection at 116–120 h after egg collection.

### Immunohistochemistry

Immunological staining was performed based on our previous methods^98^. In the present study, the following antibodies were used: sheep anti-DISC1 antibody diluted 1:50 (AF6699, R&D systems, Minneapolis, MN, USA), mouse anti-synaptotagmin diluted 1:2 (3H2 2D7, Developmental Studies Hybridoma Bank (DSHB), University of Iowa, IA, USA), mouse anti-*Drosophila* GluRIIA diluted 1:50 (8B4D2, DSHB), mouse anti-Futsch diluted 1:2 (22C10, DSHB), mouse anti-Brp^64,66^ diluted 1:20 (NC82, DSHB), anti-horseradish peroxidase protein (HRP) conjugated with fluorescein-isothiocyanate diluted 1:50 (Jackson ImmunoResearch, West Grove, PA, USA), and Alexa-conjugated secondary antibodies diluted 1:1000 (Molecular probes, Eugene, OR, USA). We captured the confocal images using a Zeiss LSM510 or LSM710 microscope.

### Quantification of NMJ structure and fluorescence intensity

To quantify synaptic phenotypes, we used the larval longitudinal muscles 6/7 in the abdominal hemisegment A2, consistent with our previously described methods^52,53^ using Image-J (http://rsb.info.nih.gov/ij/). For NMJ structural quantification (Figs. 1 and 2, Supplementary Fig. S1), we used anti-HRP and anti-synaptotagmin antibodies to label neuronal terminal and synaptic boutons, respectively. The total bouton area (μm^2^) and the number of boutons were determined based on the detected areas with the signals of anti-synaptotagmin immunoreactivity. The number of branch points was evaluated based on the axonal branch structures labeled with the pan-neuronal maker, anti-HRP immunoreactivity. For fluorescence intensity quantification (Figs. 3–5), protein expression levels were determined with Image-J based on fluorescence intensities of the control and test samples processed simultaneously in the same tube. Confocal images were captured using identical settings for samples. As an internal control, anti-HRP immunoreactivity was used. The protein expression level signal ratios in Figs. 3–5 were measured based on the fluorescence intensities of anti-GluRIIA, anti-Brp, or anti-Futsch immunoreactivity, normalized to anti-HRP immunoreactivity.

### Statistics

Statistical analysis was performed using GraphPad Prism (GraphPad Software, San Diego, CA). All data were subjected to D’Agostino–Pearson (Omnibus K2) normality test for Gaussian distribution and variance. For the dataset showing a Gaussian distribution (*p* > 0.05 in the normality tests), we performed parametric tests such as the two-tailed unpaired *t*-test and ANOVA followed by Holm–Sidak’s multiple comparisons. For datasets that failed to display a Gaussian distribution, we performed the nonparametric tests, such as the two-tailed Mann–Whitney *U* test and Kruskal–Wallis test, followed by Dunn’s multiple comparisons. Significance levels in the figures are represented as *p* < 0.05 (*), *p* < 0.01 (**), *p* < 0.001 (***), and *p* < 0.0001 (****). Data are presented as the mean ± the standard error of the mean (SEM).

## Supplementary information

Supplementary Material

## Data Availability

The data of this study are available from the corresponding author upon reasonable request.
