# Supplementary material for: FMR1 genetically interacts with DISC1 to regulate glutamatergic synaptogenesis
Source: Schizophrenia (Heidelb). 2024 Nov 27;10(1):112. doi: 10.1038/s41537-024-00532-7 (PMC11603133; doi:10.1038/s41537-024-00532-7)
Supplement: Supplementary file 1 — Supplementary Material [file 41537_2024_532_MOESM1_ESM.pdf]

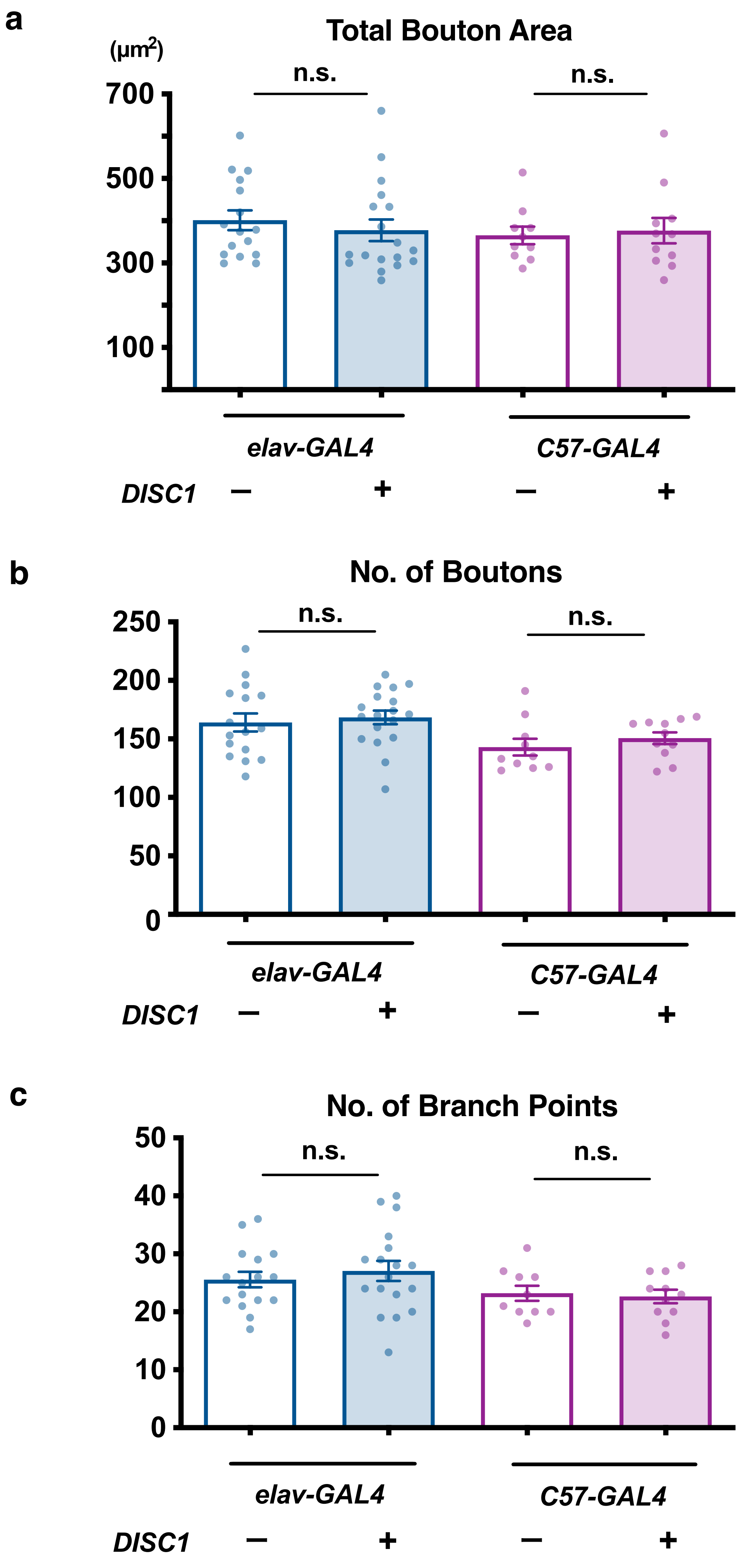
a
Total Bouton Area
(μm2)
700
500
300
100
n.s.
n.s.
elav-GAL4
C57-GAL4
DISC1
–
+
–
+
b
No. of Boutons
250
200
150
100
50
0
n.s.
n.s.
elav-GAL4
C57-GAL4
DISC1
–
+
–
+
c
No. of Branch Points
50
40
30
20
10
0
n.s.
n.s.
elav-GAL4
C57-GAL4
DISC1
–
+
–
+

Figure S1

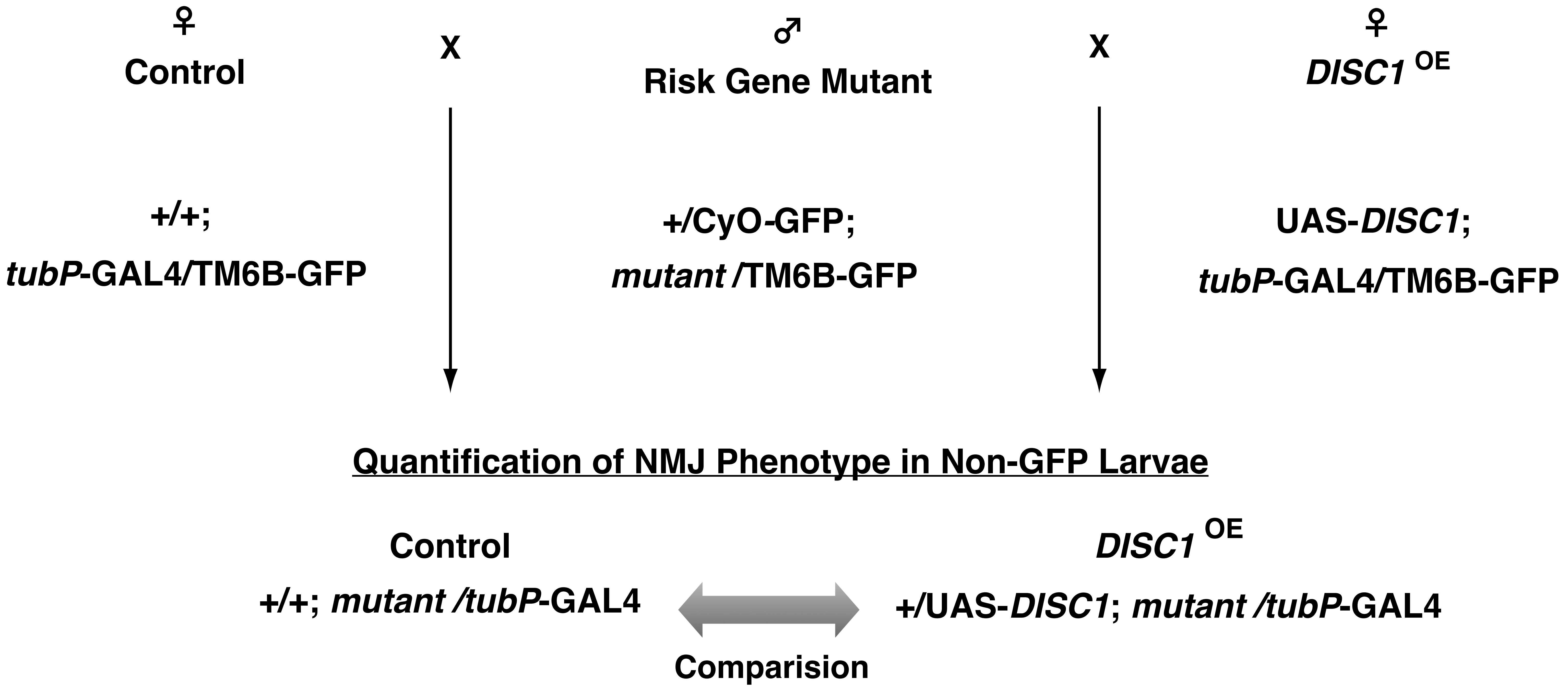
♀
Control
X
♂
Risk Gene Mutant
X
♀
DISC1 OE
+/+;
tubP-GAL4/TM6B-GFP
+/CyO-GFP;
mutant /TM6B-GFP
UAS-*DISC1*;
tubP-GAL4/TM6B-GFP
Quantification of NMJ Phenotype in Non-GFP Larvae
Control
+/+; *mutant* /*tubP*-GAL4
DISC1 OE
+/UAS-*DISC1*; *mutant* /*tubP*-GAL4
Comparision

Figure S2

Supplementary Figures

Figure S1. Analysis of NMJ morphology of pre- or post-synaptic *DISC1* overexpression. **(a)** Quantification of the NMJ morphology of the total bouton area (μm^2) in *elav-GAL4* or *C57-GAL4* larvae with (+) or without (-) DISC1 overexpression (*DISC1*^{OE}). Two-tailed Mann-Whitney *U* test (*elav-GAL4*; *DISC1*^{OE} (-) vs. *elav-GAL4*; *DISC1*^{OE} (+), $p = 0.2478$. *C57-GAL4*; *DISC1*^{OE} (-) vs. *C57-GAL4*; *DISC1*^{OE} (+), $p > 0.9999$). **(b)** Quantification of the NMJ number of boutons in *elav-GAL4* or *C57-GAL4* larvae with (+) or without (-) *DISC1*^{OE}. Two-tailed unpaired *t* test (*elav-GAL4*; *DISC1*^{OE} (-) vs. *elav-GAL4*; *DISC1*^{OE} (+), $p = 0.6504$, $t = 0.4574$, $df = 32$. *C57-GAL4*; *DISC1*^{OE} (-) vs. *C57-GAL4*; *DISC1*^{OE} (+), $p = 0.3852$, $t = 0.8889$, $df = 19$). **(c)** Quantification of the NMJ axonal branch points in *elav-GAL4* or *C57-GAL4* larvae with (+) or without (-) *DISC1*^{OE}. Two-tailed unpaired *t* test (*elav-GAL4*; *DISC1*^{OE} (-) vs. *elav-GAL4*; *DISC1*^{OE} (+), $p = 0.5075$, $t = 0.6703$, $df = 32$. *C57-GAL4*; *DISC1*^{OE} (-) vs. *C57-GAL4*; *DISC1*^{OE} (+), $p = 0.7507$, $t = 0.3224$, $df = 19$). **(a-c)** NMJs of muscles 6-7 in the second abdominal segment were stained with anti-HRP and anti-synaptotagmin. n.s., not significant. $n = 10$-18. Individual values are plotted in the graphs. Data are presented as the mean ± SEM.

Figure S2. Genetic screening of *DISC1* interactors in the fruit fly synaptogenesis. Mutant flies (*+/CyO-GFP*; *mutation*/TM6B-GFP) of the fruit fly homologue for a schizophrenia risk gene are crossed with the control (+/+; *tubP-GAL4*/*TM6B-GFP*) or the *DISC1*^{OE} (*UAS-DISC1*; *tubP-GAL4*/*TM6B-GFP*) flies. Larval NMJs of the control (*+/+; mutation/tubP-GAL4*) and *DISC1*^{OE} (*+/UAS-DISC1; mutation/tubP-GAL4*) progenies were compared. Note that while both progenies are heterozygous for the *mutation,* control flies carry the *tubP-GAL4* but not the *UAS-DISC1* transgene. Illustrated are the genetic crossings for a third chromosome mutation. Similar crossings were performed for second chromosome mutations.

Supplementary Table S1

Human Risk Genes and Fly Mutants Examined

Risk Gene	HGNC ID	Fly Homologue	Fly Mutant Allele	Fly Chromosome
ADAMTSL3	14633	CG31619	$CG31619^{DG09212}$	2
Akt1	391	Akt1	Akt^{104226}	3
ANK3	494	Ank2	$Ank2^{f02001}$	3
CACNA1C	1390	Ca-a1D	$Ca\text{-}\alpha 1D^{X10}$	2
CENTG2	16922	cenG1A	$cenG1A^{EY01217}$	2
CNTNAP2	13830	Nrx-IV	$Nrx\text{-}IV^{EY06647}$	3
CYFIP1	13759	CYFIP	$Sra\text{-}1^{EY06562}$	3
DGCR8	2847	pasha	$pasha^{EY01325}$	3
DLX1	2914	Dll	Dll^{md23}	2
DTNBP1	17328	dysbindin	$dysb^{e01028}$	3
ERBB4	3432	Egfr	$Egfr^{f2}$	2
FAM69A	32213	CG12038	$CG12038^{MB06619}$	3
FMR1	3775	Fmr1	$Fmr1^{\Delta 113M}$	3
Girdin	25523	Girdin	$Girdin^{KG07727}$	3
GRIN1	4584	Nmdar1	$Nmdar1^{DG23512}$	3
GRIN3A	16767	Ir85a	$Ir85a^{MB04613}$	3
HTR2A	5293	5-HT2	$5\text{-}HT2^{C1644}$	3
JAZF1	28917	CG12054	$CG12054^{DG06105}$	3
KCNH2	6251	sei	$sei^{HP21840}$	2
MAP2	6839	tau	tau^{MR22}	3
MAPK3	6877	rl	rl^{10a}	2
MDGA1	19267	ed	ed^{k01102}	2
MTHFR	7436	CG7650	$CG7650^{EY23633}$	3

MYO18B	18150	Mhcl	Mhcl^{NP1604}	3
NDE1	17619	nudE	nudE^{G14350}	3
NRG1	7997	vn	vn^{C221}	3
NRXN1	8008	Nrx-1	P{GSV2}GS7152	2
OLIG2	9398	tx	tx^{1}	3
OPCML	8143	CG31646	CG31646^{MB09592}	2
ORMDL3	16038	ORMDL	ORMDL^{e03591}	3
PARK2	8607	park	park^{1}	3
PLAA	9043	Plap	Plap^{d09025}	2
PRSS16	9480	CG9953	CG9953^{KG09912}	3
RGS4	10000	loco	loco^{KG02176}	3
RPL5	10360	RpL5	RpL5^{2d2}	2
SEMA3C	10725	Sema-2a	Sema-2a^{03021}	3
SLC18A1	10934	Vmat	Vmat^{SH0459}	2
SHOX	10853	CG34367	CG34367^{f00117}	2
SRR	14398	CG8129	CG8129^{c04459}	3
TBX6	11605	Dorsocross 1	Doc1^{MB02443}	3
		Dorsocross 2	Doc2^{MB09116}	3
TCF4	11634	da	da^{1}	2
TPH1	12008	Trh	Trh^{c01440}	3
TRAX	12380	Trax	Trax^{G18534}	3

Supplementary Table S2

DISC1 Binding Proteins Targeted by FMRP

Genes *1	HGNC ID	Encoded Protein	Fly Homolog *2	Reference *3
AGTPBP1	17258	Cytosolic carboxypeptidase 1	*CG31019*	Camargo 2007
AKAP9	379	A-kinase anchor protein 9	*cp309*	
APP	620	Amyloid beta A4 protein	*Appl*	Young-Pearse 2010
ATF7IP	20092	Activating transcription factor 7-interacting protein 1	*wde*	Morris 2003
DCTN1	2711	Dynactin subunit 1	*Gl*	Camargo 2007
DNCH1	2961	Cytoplasmic dynein 1 heavy chain 1	*Dhc64C*	
DPYSL2	3014	Dihydropyrimidinase-related protein 2	*CRMP*	
DST	1090	Dystonin	*shot*	
EEF2	3214	Elongation factor 2	*Eef2*	
FBXO41	29409	F-box only protein 41	-	
GNB1	4396	Guanine nucleotide-binding protein G(I)/G(S)/G(T) subunit beta-1	*Gbeta13F*	
GSK3B	4617	Glycogen synthase kinase-3 beta	*gskt*	Mao 2009
ITSN1	6183	Intersectin-1	*Dap160*	Morris 2003
KALRN	4814	Kalirin	*trio*	Camargo 2007
KIF3C	6321	Kinesin-like protein KIF3C	*Klp68D*	
MACF1	13664	Microtubule-actin cross-linking factor 1, isoforms 1/2/3/5	*shot*	
MAP1A	6835	Microtubule-associated protein 1A	*Futsch*	Morris 2003
MYT1L	7623	Myelin transcription factor 1-like protein	*CG43689*	Camargo 2007
OLFM1	17187	Noelin	*CG6867*	
PDE4B	8781	cAMP-specific 3',5'-cyclic phosphodiesterase 4B	*dnc*	
PPM1E	19322	Protein phosphatase 1E	*CG10376*	
SPARCL1	11220	SPARC-like protein 1	*BM-40-SPARC*	
SPTAN1	11273	Spectrin alpha chain, non-erythrocytic 1	*alpha-Spec*	
SPTBN1	11275	Spectrin beta chain, non-erythrocytic 1	*beta-Spec*	
SYNE1	17089	Nepsin 1	*Nepsin*	Morris 2003
TNIK	30765	TRAF2 and NCK-interacting protein kinase	*msn*	Camargo 2007
TNKS	11941	Tankyrase-1	*tankyrase*	
TRIO	12303	Triple functional domain protein	*trio*	
ZNF365	18194	Protein ZNF365	-	

*1. DISC1 binding protein genes targeted by FMRP（Darnell et al., 2011）.

*2. Identified with Integrative Ortholog Prediction Tool (DIOPT) at the Drosophila RNAi Screening Center.

*3. References for DISC1 binding.

Supplementary References

1. Camargo et al., Disrupted in Schizophrenia 1 Interactome: evidence for the close connectivity of risk genes and a potential synaptic basis for schizophrenia. *Mol. Psychiatry* **12**, 74-86 (2007).
2. Darnell et al., FMRP stalls ribosomal translocation on mRNAs linked to synaptic function and autism. *Cell* **146**, 247-261 (2011).
3. DRSC Integrative Ortholog Prediction Tool. http://www.flyrnai.org/cgi-bin/DRSC_orthologs.pl
4. Hu et al., An integrative approach to ortholog prediction for disease-focused and other functional studies. *BMC Bioinform.* **12**, 357 (2011).
5. Mao et al., Disrupted in schizophrenia 1 regulates neuronal progenitor proliferation via modulation of GSK3beta/beta-catenin signaling. *Cell* **136**, 1017-1031 (2009).
6. Morris et al., DISC1 (Disrupted-In-Schizophrenia 1) is a centrosome-associated protein that interacts with MAP1A, MIPT3, ATF4/5 and NUDEL: regulation and loss of interaction with mutation. *Hum. Mol. Genet.* **12**, 1591-1608 (2003).
7. Young-Pearse et al., Biochemical and functional interaction of disrupted-in-schizophrenia 1 and amyloid precursor protein regulates neuronal migration during mammalian cortical development. *J. Neurosci.* **30**, 10431–10440 (2010).
